# The Anti-Inflammatory Actions of Auricular Point Acupressure for Chronic Low Back Pain

**DOI:** 10.1155/2015/103570

**Published:** 2015-06-11

**Authors:** Wei-Chun Lin, Chao Hsing Yeh, Lung-Chang Chien, Natalia E. Morone, Ronald M. Glick, Kathryn M. Albers

**Affiliations:** ^1^School of Nursing, University of Pittsburgh, Pittsburgh, PA 15213, USA; ^2^Department of Biostatistics, University of Texas, School of Public Health at San Antonio Regional Campus, University of Texas Health Science Center at San Antonio Regional Campus, Research to Advance Community Health Center, Houston, TX 77025, USA; ^3^Division of General Internal Medicine, Department of Medicine, School of Medicine, University of Pittsburgh and Geriatric Research Education and Clinical Center, Veterans Administration Pittsburgh Healthcare System, Pittsburgh, PA 15213, USA; ^4^Departments of Psychiatry, Physical Medicine, and Rehabilitation, School of Medicine, University of Pittsburgh, Pittsburgh, PA 15213, USA; ^5^Department of Neurobiology, University of Pittsburgh, Pittsburgh, PA 15213, USA

## Abstract

*Background.* Auricular point acupressure (APA) is a promising treatment for pain management. Few studies have investigated the physiological mechanisms of APA analgesics.* Method.* In this pilot randomized clinical trial (RCT), a 4-week APA treatment was used to manage chronic low back pain (CLBP). Sixty-one participants were randomized into a real APA group (*n* = 32) or a sham APA group (*n* = 29). Blood samples, pain intensity, and physical function were collected at baseline and after 4 weeks of treatment.* Results.* Subjects in the real APA group reported a 56% reduction of pain intensity and a 26% improvement in physical function. Serum blood samples showed (1) a decrease in IL-1*β*, IL-2, IL-6, and calcitonin gene-related peptide [CGRP] and (2) an increase in IL-4. In contrast, subjects in the sham APA group (1) reported a 9% reduction in pain and a 2% improvement in physical function and (2) exhibited minimal changes of inflammatory cytokines and neuropeptides. Statistically significant differences in IL-4 and CGRP expression between the real and sham APA groups were verified.* Conclusion.* These findings suggest that APA treatment affects pain intensity through modulation of the immune system, as reflected by APA-induced changes in serum inflammatory cytokine and neuropeptide levels.

## 1. Introduction

Chronic low back pain (CLBP) is a major health problem worldwide and is associated with high medical costs, lost productivity, and long-term disability [1–3]. Although various standard pharmacologic and nonpharmacologic treatments have been proposed to alleviate CLBP, their effectiveness is limited [4]. The growing prevalence of CLBP and the limited treatments available underscore an increasing need for complementary therapies, which is reflected by more than one-third of adults with low back pain in the United States who have been treated by an integrative medicine provider over the past decade [5–7].

Auricular point acupressure (APA) is a treatment method similar to acupuncture that may allow improved management of CLBP pain. APA is one form of acupressure in traditional Chinese medicine (TCM) in which specific acupoints on the ear are stimulated without the use of needles [8, 9]. Auricular therapy was modified and updated by Dr. Nogier, the “father of auriculotherapy,” in the 1950s [10]. Since then, the World Health Organization has come to recognize auricular therapy as a form of microacupuncture that can affect the entire body [11].

Previous studies using APA for managing pain relief in CLBP have been promising [12–16]. For example, APA provided immediate relief for CLBP (i.e., 40% reduction in pain intensity after 1 day of APA) [13] and even greater and lasting effects on reducing CLBP (i.e., 75% pain relief and 45% better physical function after a 4-week treatment—both statistically significant compared to a sham APA group) [12]. Additionally, APA is a feasible intervention for older adults [14, 16]. In a study of 37 older adults with CLBP who received 4 weeks of APA, a significant reduction (i.e., 41% for those (*n* = 19) in the real APA group and 5% for the sham APA group (*n* = 18) in worst pain from baseline to the end of the treatment was reported [14]. Improved physical function was also achieved (i.e., the Roland Morris Disability Questionnaire [RMDQ] score decreased in the real APA group by 29% and was unchanged in the sham APA group) [14].

Increasing evidence supports immune activation in the etiology and progression of CLBP [17–19]. Immune biomarkers assessed typically include proinflammatory and anti-inflammatory cytokines and neuropeptides [20]. Changes in proinflammatory cytokines, such as IL-1*β*, IL-2, IL-6, and TNF-*α*, have been linked to alteration in pain signaling pathways [21]. Although the relationship between disk degeneration and CLBP remains unclear, evidence suggests that IL-1 and IL-6 may contribute to a local enhancement of pain by promoting matrix degradation [22, 23]. Moreover, IL-1, IL-6, and TNF-*α* may be associated with the expression of matrix metalloproteinases, which can lead to the herniation of intervertebral disks [24, 25]. These findings suggest a possible role for cytokines, in addition to chronic inflammation, among patients with CLBP.

In contrast, anti-inflammatory cytokines, such as IL-4 and IL-10, may inhibit the proinflammatory cytokine response [26]. Anti-inflammatory cytokines, such as TNF, IL-1, and IL-6, are produced by activated macrophages and monocytes and can act to inhibit the synthesis of proinflammatory cytokines [27]. IL-4 and IL-10 also suppress Th1 cells from releasing proinflammatory cytokines and inducing B-lymphocyte differentiation [27].

The level of calcitonin gene-related peptide (CGRP) has also been linked to pain signaling in CLBP [28]. In humans, CGRP exists in two forms: *α*-CGRP and *β*-CGRP [29]. Each binds to a G-protein-coupled receptor, and this activation is thought to produce long-lasting modifications of neurotransmission [30]. CGRP is also a potent vasodilator [31]. Interestingly, IL-1*β* stimulates the release of CGRP, while IL-6 stimulates the synthesis of CGRP in sensory neurons [32]. Cells of the immune system also synthesize *β*-endorphin [33]. T-lymphocytes, B-lymphocytes, monocytes, and macrophages have been shown to contain endorphins during inflammatory states [33].

How APA induces changes in cytokines and endorphins in immune and neuronal cell types is still unclear. One explanation is that pain and neuronal excitability impact a neural immune pathway that interconnects the ear microsystem and the somatotopic brain [8]. Neurophysiological connections between ear acupoints and the human CNS have been supported by fMRI studies [34]. Stimulation of acupoints is thought to cause vasodilatation through release of *β*-endorphin, which elicits either short-term analgesic effects or neuropeptide-induced anti-inflammatory cytokines for long-term effects [35–37].

To examine the underlying biological mechanisms of APA in pain relief, we previously measured serum levels of various cytokines in a prospective 4-week RCT of APA therapy for CLBP [38]. In this pilot study, subjects in the real APA group who reported a 70% reduction in worst pain intensity had changes in serum cytokine levels [38]. In particular, IL-2 and TNF-*α* decreased, and IL-10 increased, in subjects receiving APA. In contrast, subjects of the sham APA group reported only a 29% reduction in pain, and their levels of cytokines exhibited a different profile: IL-2, IL-4, and TNF-*α* decreased and IL-1*β*, IL-6, and IL-10 increased [38]. Among all subjects, levels of IL-1*β*, IL-2, IL-6, and IL-10 were associated with the worst pain intensity score [38]. This prior pilot study had a small sample size and lacked significance, necessitating further research to determine effect size to use for power calculation. The primary aims of this pilot study were to (1) collect more data for effect size and power calculation, (2) confirm our previous findings of a differential response between groups, which reflected the anti-inflammatory effect of the real APA intervention, and (3) investigate the association between biomarker change and clinical outcome.

## 2. Methods

Complete details of the study design, sample, and data collection are provided in our previous manuscripts [12, 14], and participant recruitment began after approval by University of Pittsburgh, Institutional Review Board. For this study, we increased the sample size and measured the circulating levels of inflammatory cytokines (i.e., IL-1*β*, IL-2, IL-4, IL-6, IL-10, and TNF-*α*) and *β*-endorphin and CGRP. Levels of these compounds were measured in subjects who received treatment in either a real APA group or a sham APA group. Measures were correlated with clinical outcomes (including worst pain intensity and physical function) for participants who completed the baseline assessment (pre-APA treatment) and the 4-week APA treatment (post-APA treatment). The real APA group had 32 participants and the sham APA group had 29 (including 19 participants for which data had been published) [38]. For these 61 participants, 27% were receiving other treatments (pain medication, *n* = 15; chiropractor, *n* = 1; massage, *n* = 1), 62% (*n* = 38) were not receiving treatment, and 11% (*n* = 6) had never received any treatment related to CLBP. All participants suffered CLBP, which, for the purpose of this study, was defined as low back pain occurring for at least 3 months with an average pain intensity score of 4 or greater on a 0–10-point numerical scale for 1 week prior to enrollment. The majority of the participants' medical diagnoses were osteoarthritis (44%, *n* = 27) and spinal stenosis (39%, *n* = 24). Demographic background information for the participants appears in Table 1. The mean age of the participants was 58.03 years (SD = 17.28) for the real APA group and 62.80 years (SD = 14.75) for the sham APA group.

### 2.1. Auricular Point Acupressure Treatment Protocol

The APA intervention included one treatment per week for 4 consecutive weeks. Auricular points on the ears of participants were detected with an electrical acupoint finder, which measures auricular cutaneous resistance to identify the potential acupoints for treatment. Using TCM and Chinese ear acupoint maps, the acupoints selected for the real APA group included three for alleviating stress and pain (i.e.,* shenmen*,* sympathetic point*, and* nervous subcortex*) and one corresponding to the anatomical site (i.e.,* lower back*) [8]. The acupoints selected for the sham APA group were located away from the site where the participant was experiencing pain and included* stomach*,* mouth*,* duodenum*, and* kidney*. In our published APA protocol [12, 14], participants were told to press/stimulate the seeds taped to the acupoints on their ears at least 3 times per day for 3 minutes each time. The seeds and tape were removed at the end of the 5th day each week to insure baseline sensitivity to the site prior to the next treatment. The primary endpoint was the measure of pain intensity and physical function after completion of the 4-week APA. Participants in the sham APA group, who were blinded to this assignment, were provided the opportunity to receive real APA treatment after completing all assessments.

### 2.2. Data Collection Procedure

Blood (10 mL) was collected from participants in both treatment groups in a red-top vacutainer, using standard phlebotomy procedures. Blood was drawn by a trained nurse before the APA treatment (baseline), once a week for the 4 weeks of APA treatment, after the 4-week APA treatment, and at the 1-month follow-up. Due to budget limitations, data were only obtained for the pre-APA (baseline) and post-APA treatment (after the 4-week APA treatment).

### 2.3. Blood Extraction

Tubes containing blood samples were labeled with the participant's ID number and time of collection, placed on a level rack at room temperature, and left undisturbed for 1.5 hours. After the tubes were centrifuged at 1,500 rpm for 10 minutes, the serum was transferred into 0.5 mL polypropylene microcentrifuge tubes and stored at −80°C until assayed.

### 2.4. Inflammatory Biomarker Testing

Luminex cytokine analysis (xMAP, Multiplexed or Multianalyte Platform, Austin, Texas) was used to measure IL-1*β*, IL-2, IL-6, IL-4, IL-10, and TNF-*α*. Serum was assayed in the Luminex Core Facility at the University of Pittsburgh Cancer Institute. xMAP technology uses polystyrene microspheres internally dyed with varying ratios of two spectrally distinct fluorophores to create a family of 100 differentially spectrally addressed bead sets. Each bead set was conjugated with a capture antibody specific for a unique target analyte and allowed to react with the serum sample. Beads were washed and secondary (or detection) antibodies were added to a microtiter plate well to perform a capture sandwich immunoassay. The bead suspension was analyzed using a fluorometric array reader with two fluorescence readings obtained for each bead. One reading indicated whether or not a bead was a member of one of the 100 possible sets. The other reading corresponded to the amount of fluorescent dye, typically phycoerythrin (PE), bound to the detection antibody in the assay. The amount of PE fluorescence was proportional to the amount of analyte captured in the immunoassay. Bio-Plex Manager software was used to correlate readouts from each bead set with the assay reagent coupled with the bead set. Results were extrapolated to a standard curve that was used to quantify each analyte in the sample. The xMAP assay used in this study measured a maximum of 100 analytes in a 96-well microplate in 1 hour.

Serum levels of *β*-endorphin and CGRP were determined using commercial ELISA kits (MyBioSource, San Diego, CA) according to the manufacturer's instructions. The level of sensitivity and precision of each assay were as follows: *β*-endorphin = 2.59 ng/L (intra-assay coefficient of variation [CV] <10%, interassay CV < 12%) and CGRP = 1.12 ng/L (intra-assay CV < 10%, interassay CV < 12%).

### 2.5. CLBP Clinical Outcomes

CLBP clinical outcomes included assessments of pain intensity and physical functioning.* Worst pain* was an individual item from the Brief Pain Inventory short form (BPI-sf) [39]. From baseline, on a 0–10 numerical scale, the cut-off point of 10–20% was rated as “minimally important,” 30% or greater as “moderately important,” and 50% or more as “substantial” pain intensity change [40].* Physical functioning* was measured by the Roland-Morris Disability Questionnaire (RMDQ) [41]. The RMDQ is a 24-item measure to assess the impact of back-related pain on daily functioning. Participants selected “yes” or “no” for statements related to their physical function. The total score ranged from 0 (no disability) to 24 (maximum disability). The RMDQ is a reliable, valid, and sensitive measure that has demonstrated substantial construct validity [41, 42]. A RMDQ reduction of 30% or greater is rated as “minimally clinically important” [43].

### 2.6. Data Analysis

Descriptive analysis was used to display the outcomes measures (including cytokine, neuropeptide, pain intensity, and physical functions). Because the sample size was approximately 30 per group, independent two-sample *t*-test was used to compare the mean change from pretreatment to posttreatment between the real and sham APA groups. Pearson product-moment correlation coefficient (*r*) was used to examine the linear association of the changes from pretreatment to posttreatment in cytokine level and clinical outcomes (i.e., pain intensity and physical functions). Significance was set at a *P* value < 0.05. All data analyses were performed using SAS software, version 9.2 [44].

## 3. Results

### 3.1. Characteristics of Biomarkers and Clinical Outcomes

Table 2 presents the descriptive characteristics of cytokine, CGRP and *β*-endorphin levels, and clinical outcome at pre- and post-APA treatment. The biomarker data were skewed, and the median should be reported for descriptive characteristics. We also present the mean and standard deviation to understand the trend of change to guide the design of a future study.

Table 3 lists the mean changes from pre- to post-APA treatment for each biomarker. Statistically significant differences between the real APA group and the sham APA group were determined for IL-4 (mean difference = 1.33, SD = 2.49, and *P* = 0.05) and CGRP (mean difference = −8.87, SD = 14.19, and *P* = 0.04) levels. Changes in other biomarker levels did not reach statistical significance between the two groups. For clinical outcomes, pain intensity measures showed a statistically significant improvement among individuals in the real APA group (−3.66, SD = 2.78) compared to participants in the sham APA group (−0.79, SD = 2.46), resulting in a −2.86 (SD = 3.63) difference between the real and sham APA groups (*P* value < 0.01). However, no statistically significant finding was found for physical function.

Further comparison between groups revealed proinflammatory cytokine and CGRP to display a trend of mean reduction from pre- to post-APA treatment (i.e., −1.33 in IL-1*β*, −1.24 in IL-2, −2.18 in IL-6, and −6.19 in CGRP) for the real APA group. In the sham APA group, proinflammatory cytokines also displayed a decreasing trend in mean changes yet with smaller magnitudes compared to the real APA group (i.e., −0.39 in IL-1*β*, −0.39 in IL-2, −1.28 in IL-6, and −5.61 in TNF-*α*). The anti-inflammatory cytokine IL-4 increased from pre- to post-APA treatment for the participants in the real APA group; however, IL-4 decreased in the sham APA group. IL-10 and *β*-endorphin decreased for both groups. A statistically significant change from pre- to post-APA treatment for all biomarkers was not observed. For clinical outcomes, the mean pain intensity score and physical function score exhibited a statistically significant change from pre- to post-APA treatment in the real APA group (*P* < 0.01), which indicates that participants in the real APA group experienced a marked reduction in pain intensity (56%) and improved physical function (26%). The effect sizes for each biomarker are presented in Table 3.

### 3.2. Correlation of Cytokines, Neuropeptides, and Clinical Outcomes

Pearson correlation coefficients for biomarkers and clinical outcomes are presented in Table 4 for pre-APA treatment (upper triangular region) and mean changes from pre- to post-APA treatment (lower triangular region) for the real APA group—data for sham APA group participants is available upon request. In the pre-APA treatment group, proinflammatory cytokines (i.e., IL-1*β*, IL-2, and IL-6) had strong linear associations (*r* > 0.7) among other cytokines in this category (i.e., IL-1*β*/IL-2, *r* = 0.98; IL-1*β*/IL-6, *r* = 0.85; and IL-2/IL-6, *r* = 0.82). Moderate linear associations (0.3 ≤ *r* ≤ 0.7) were found between IL-4 and IL-10 in the category of anti-inflammatory cytokines (*r* = 0.45). There was also a moderate linear association between cytokines in the proinflammatory and anti-inflammatory categories. Additionally, a negative relationship was found between CGRP and physical function (*r* = −0.46), and a moderate linear association between the mean change in pain scores and physical function (*r* = 0.52) was observed. The mean change score of CGRP was moderate when associated with IL-1*β* (*r* = 0.41) and IL-2 (*r* = 0.42). The correlations among the mean score changes of biomarkers and clinical outcomes were weak.

## 4. Discussion

The present study not only expands on data presented in a previous pilot study [38], but also further examines whether or not serum cytokine and/or neuromodulator levels change in response to APA treatment for CLBP. Participants in the real APA group reported a mean 56% reduction in pain intensity and a mean 26% improvement in physical function at the completion of the 4-week APA regimen. We also observed decreases in serum proinflammatory cytokines (i.e., IL-1*β*, IL-2, and IL-6) and CGRP and increases in IL-4, an anti-inflammatory cytokine. The sham APA group exhibited a 9% pain reduction, 2% improved physical function, and decreased levels of IL-1*β*, IL-2, IL-6, and IL-4; CGRP and *β*-endorphin levels also increased. Additionally, the level of IL-4 was significantly higher and CGRP was lower in the real APA groups compared to levels in the sham APA group. These results indicate preliminary associations among CGRP, IL-4, IL-1*β*, IL-2, IL-6, and the pain intensity score, which suggest a pathophysiological mechanism underlies the APA analgesic effect on CLBP.

These outcomes are consistent with previous studies reporting increased levels of proinflammatory cytokines in CLBP [45–47]. For example, a cross-sectional study of 23 patients with CLBP diagnosed with herniated intervertebral disks and 10 healthy controls showed statistically significant increases in concentrations of IL-6 and TNF-*α*, but not IL-1*β*, in patients with CLBP [48]. Another study of 94 patients diagnosed with chronic neuropathic, nociceptive, or mixed pain for more than 6 months and six healthy controls reported a positive correlation between increased cytokine concentration, including IL-1*β*, IL-6, and TNF-*α*, and increased pain severity [49].

It has been proposed that peripheral immune responses lead to the activation of discrete circuitries within the central nervous system via both hematogenous and neural pathways, facilitating changes known as sickness responses [50]. Cytokines, as sickness inducing agents, are recognized as key mediators of immune-to-brain communication that facilitate pain [50]. The medulla-to-spinal cord limb of this pathway is proposed to modulate release of neurotransmitters that activate spinal cord glia and enhance pain [50]. Blocking proinflammatory cytokines such as IL-1*β* and IL-6 by administration of specific antagonists can prevent the generation of sickness responses induced by peripheral immune challenges [50]. Moreover, proinflammatory cytokines administered peripherally in the absence of peripheral immune challenge are sufficient to induce sickness responses [51, 52]. Our results show a decrease in proinflammatory cytokines (including IL-1*β*, IL-2, and IL-6), an increase in anti-inflammatory cytokine (i.e., IL-4), and a decrease in CGRP between pre- and post-APA treatments. A moderate correlation was observed between pain intensity change and IL-1*β* and IL-2 changes. Based on these findings, we hypothesize that APA therapy may exhibit anti-inflammatory efficacy in CLBP in two ways: (1) downregulation of proinflammatory cytokines (i.e., IL-1*β*, IL-2, and IL-6) and upregulation of anti-inflammatory cytokines (i.e., IL-4) and (2) downregulation of proinflammatory neuropeptides (i.e., CGRP).

An unexpected finding in this study was the decrease in *β*-endorphin. *β*-endorphin produces analgesia by (1) binding opioid receptors at both presynaptic and postsynaptic nerve terminals in the peripheral nervous system [53] and (2) inhibiting neuronal firing of somatosensory fibers, especially those involved in nociception [54]. Studies to identify mechanisms of acupuncture-mediated analgesia also suggest a role for endogenous opioid peptides, such as *β*-endorphin [55]. Acupuncture is thought to cause vasodilation by releasing *β*-endorphin and, in so doing, elicit short-term analgesic effects [35–37]. The relationship of increased *β*-endorphin and reduced pain is based on primarily animal studies [56]; we lack empirical studies of this relationship in humans. Additionally, various testing procedures are used to measure *β*-endorphin levels in serum that may induce variability in measurement [57]. In this study, blood was collected only one time to determine the level of *β*-endorphin. After collection, blood was kept at room temperature for 1.5 hours, which deviates from the optimum conditions for *β*-endorphin (i.e., blood is placed on ice immediately after collection, serum separation occurs, and then samples are frozen within 1 hour after collection) [58]. Additionally, we did not record possible confounding variables that may impact *β*-endorphin level, such as smoking, alcohol consumption, medication, and stress. Additional statistical analysis of the cross-reaction of *β*-endorphin with other cytokines could reveal novel interactions.

Despite the strengths of the study as an RCT with a sham control group, this study has limitations. First, we were unable to determine the biological actions of the APA intervention due to the small sample size and complexity of pathophysiology, which involves factors that cross-react within biomarkers. However, we did identify changes in biological biomarker patterns. For example, CGRP decreased in the real APA group while it increased in the sham APA group. Likewise, IL-4 increased in the real APA group and decreased in the sham APA group. Second, chronic pain pathophysiology is complicated. Although we attempted to include most related biomarkers, we are unable to define an underlying biological mechanism of APA therapy on CLBP. Lastly, we did not collect confounding variables that may cross-react with biomarkers, including medication use and stress. Nevertheless, (1) expression of proinflammatory cytokines such as IL-1*β*, IL-2, and IL-6 decreased and (2) the expression of the anti-inflammatory cytokine IL-4 increased in the real APA group after 4 weeks of APA treatment, both suggesting the interaction of APA therapy and neural-immune signaling. Moreover, CGRP decreased after 4 weeks of APA treatment, and this decrease may impact the sickness response and alleviate symptoms of CLBP. These preliminary findings warrant a larger clinical trial that could further elucidate the biological mechanism of auricular therapy for pain relief.

## 5. Conclusion

The change in cytokine and neuropeptide levels among the participants who received APA treatment indicates that APA could mediate the expression of inflammatory cytokines and neuropeptides and thus decrease pain intensity. We were able to verify statistically significant differences in IL-4 and CGRP expression between real and sham APA groups. However, the biological mechanism for chronic pain is complicated by pathophysiology, involving factors that cross-react with biomarkers, including medication use and stress. Nevertheless, our findings strongly suggest that pain relief and improved physical function in patients with CLBP experienced through APA treatment may be modulated by the level of inflammatory cytokines (i.e., IL-1*β*, IL-2, IL-4, and IL-6) and neuropeptides (i.e., CGRP). Our findings warrant additional research, which could include larger-scale studies to determine the underlying biological mechanism linking APA, cytokine/neuropeptide levels, and pain relief.

## Figures and Tables

**Table 1:** Demographic characteristics of the participants (*n* = 61).

	Mean (SD) or *n* (%)		*P*/*χ* ^2^
	Real (*n* = 30)	Sham (*n* = 31)	
Age			
Mean (SD)	61 (17.44) (20–82)	66 (16.04) (21–90)	0.91
Gender, *n* (%)			
Male	10 (33%)	10 (32%)	0.93
Female	20 (67%)	21 (68%)	
Race/ethnicity, *n* (%)			
White	26 (87%)	25 (81%)	0.73
Black/African American	4 (13%)	6 (19%)	
Marital status, *n* (%)			
Married or living with partner	14 (47%)	13 (42%)	0.78
Divorced or widowed	10 (33%)	11 (36%)	
Never married	6 (20%)	5 (16%)	
Employment situation			
Working (full time)	6 (20%)	4 (13%)	0.67
Working (part time)	2 (7%)	2 (7%)	
Not employed	4 (13%)	6 (19%)	
Retired	15 (50%)	14 (45%)	
Others	3 (10%)	5 (16%)	
Education level			
≤10th grade	2 (6%)	2 (6%)	0.62
High school	5 (17%)	4 (13%)	
Technical or vocational school	2 (6%)	2 (6%)	
College and/or graduate	21 (71%)	21 (69%)	
Missing		2 (6%)	
Estimated income before taxes			
Less than $10,000	5 (17%)	7 (23%)	0.25
$10,000 to $19,999	2 (6%)	5 (16%)	
$20,000 to $39,999	8 (27%)	2 (6%)	
$40,000 to $59,000	6 (20%)	7 (23%)	
$60,000 to $100,000	3 (10%)	5 (16%)	
More than $100,000	3 (10%)	1 (3%)	
Missing	3 (10%)	4 (13%)	
Medical diagnosis related to back pain			
Osteoporosis	3 (10%)	4 (13%)	
Osteoarthritis	9 (30%)	9 (29%)	
Scoliosis	3 (10%)	5 (16%)	
Kyphosis	1 (3%)	0 (0%)	
Disc herniation	4 (14%)	7 (23%)	
Spinal stenosis	6 (20%)	13 (42%)	
Spondylitis	1 (3%)	0 (0%)	
Spondylosis	3 (10%)	0 (0%)	
Current pain medication use			
Yes	13 (43%)	14 (45%)	
No	17 (57%)	17 (55%)	0.89

**Table 2:** Descriptive characteristics of participants receiving APA treatment.

Outcomes	Pre-APA						Post-APA				
		Mean	SD	Median	Q1	Q3	Mean	SD	Median	Q1	Q3
Proinflammatory cytokines											
IL-1*β*	APA	16.17	11.38	12.50	9.00	19.50	14.83	10.00	12.00	8.00	15.00
	Sham	17.70	21.14	11.00	9.00	16.00	17.30	22.25	11.00	9.00	15.00
IL-2	APA	17.73	11.53	13.00	10.00	21.00	16.48	9.99	13.00	10.00	20.00
	Sham	21.29	30.34	11.50	9.50	18.50	20.89	34.39	11.75	8.50	17.00
IL-6	APA	33.28	18.47	26.50	22.00	39.00	31.10	18.09	24.50	20.00	31.00
	Sham	36.48	25.82	28.00	22.00	34.00	35.21	31.80	28.50	20.50	36.00
TNF-*α*	APA	111.68	44.13	101.50	86.50	129.50	114.23	46.60	107.00	86.00	127.00
	Sham	127.41	51.58	122.00	105.75	161.75	121.80	46.43	121.50	93.00	153.50
Anti-inflammatory cytokines											
IL-4	APA	19.78	17.86	14.00	12.00	16.00	20.18	19.35	14.00	12.00	17.50
	Sham	16.61	8.96	13.75	11.75	19.25	15.68	8.20	13.00	11.50	17.00
IL-10	APA	29.83	12.73	26.00	23.00	33.00	27.55	9.82	26.00	22.00	29.00
	Sham	32.56	14.77	29.00	24.00	34.00	31.19	13.44	28.00	22.50	32.00
Neuropeptides											
CGRP	APA	42.04	53.99	14.34	3.24	59.57	35.85	44.32	9.36	3.77	61.64
	Sham	36.74	31.59	31.10	6.40	65.05	39.43	35.85	30.43	4.34	67.58
*β*-endorphin	APA	138.21	47.95	131.28	107.55	146.74	129.54	42.57	117.40	103.52	135.99
	Sham	121.81	35.29	121.77	104.88	135.86	113.92	23.88	113.52	96.08	135.23
Clinical outcomes											
Pain intensity	APA	6.31	1.93	6.00	5.00	7.00	2.66	2.01	2.00	1.00	4.00
	Sham	6.07	1.71	6.00	5.00	7.00	5.28	2.37	5.00	4.00	7.00
Function	APA	7.43	5.61	6.00	3.00	11.00	4.77	5.20	4.00	1.00	5.50
	Sham	9.43	5.02	10.00	6.50	13.00	9.00	5.76	10.00	3.00	14.00

*Note*. SD: standard deviation; Q: quantile; APA: auricular point acupressure; CGRP: calcitonin gene-related peptide.

**Table 3:** Mean changes from pre- to post-APA treatment of biomarkers and clinical outcomes.

Cytokine change after treatment	Real APA group	Sham APA group	Difference	*P* value	Effect size^∗^
	Mean (SD)	Mean (SD)	Mean (SD)		
Proinflammatory cytokines					
IL-1*β*	−1.33 (5.00)	−0.39 (2.09)	−0.94 (3.88)	0.35	−0.24
IL-2	−1.24 (6.51)	−0.39 (5.34)	−0.85 (5.98)	0.58	−0.14
IL-6	−2.18 (9.68)	−1.28 (16.19)	−0.90 (13.58)	0.80	−0.07
TNF-*α*	2.55 (24.39)	−5.61 (22.96)	8.16 (23.71)	0.20	0.34
Anti-inflammatory cytokines					
IL-4	0.40 (2.46)	−0.93 (2.52)	1.33 (2.49)	0.05	0.53
IL-10	−2.28 (7.22)	−1.38 (7.07)	−0.90 (7.15)	0.63	−0.13
Neuropeptides					
CGRP	−6.19 (17.91)	2.69 (8.41)	−8.87 (14.19)	0.04	−0.63
*β*-endorphin	−8.67 (21.57)	−7.88 (24.49)	−0.79 (12.07)	0.90	−0.03
Clinical outcomes					
Pain	−3.66 (2.78)	−0.79 (2.46)	−2.86 (2.63)	<0.01	−1.09
Physical function	−2.86 (3.97)	−0.43 (4.52)	−0.43 (3.99)	0.06	−0.11

*Note*. SD: standard deviation; APA: auricular point acupressure; CGRP: calcitonin gene-related peptide.

^∗^The effect size is calculated from Cohen's *d*.

**Table 4:** Pearson correlation coefficients among biomarkers and clinical outcomes.

Biomarkers	Proinflammatory cytokine				Anti-inflammatory cytokine		Neuropeptides		Clinical outcomes	
	IL-1*β*	IL-2	IL-6	TNF-*α*	IL-4	IL-10	CGRP	*β*-endorphin	Pain	Physical function
IL-1*β*	—	0.98^∗^	0.85^∗^	0.32^∗^	0.37^∗^	0.20^∗^	0.15^∗^	0.02^∗^	0.05^∗^	−0.02^∗^
IL-2	0.84^#^	—	0.82^∗^	0.39^∗^	0.29^∗^	0.17^∗^	0.12^∗^	−0.04^∗^	0.06^∗^	0.02^∗^
IL-6	0.54^#^	0.57^#^	—	0.29^∗^	0.45^∗^	0.26^∗^	0.06^∗^	0.02^∗^	0.06^∗^	0.09^∗^
TNF-*α*	0.15^#^	0.31^#^	0.33^#^	—	0.04^∗^	0.31^∗^	0.06^∗^	−0.18^∗^	0.14^∗^	0.25^∗^
IL-4	0.44^#^	0.42^#^	0.25^#^	0.28^#^	—	0.45^∗^	0.06^∗^	−0.16^∗^	−0.10^∗^	−0.04^∗^
IL-10	0.33^#^	0.40^#^	0.48^#^	0.53^#^	0.30^#^	—	0.30^∗^	−0.21^∗^	−0.13^∗^	−0.27^∗^
CGRP	0.41^#^	0.42^#^	0.05^#^	0.08^#^	0.13^#^	0.04^#^	—	0.14^∗^	−0.25^∗^	−0.46^∗^
*β*-endorphin	0.02^#^	0.00^#^	0.32^#^	0.01^#^	−0.16^#^	−0.18^#^	0.076^#^	—	0.06^∗^	−0.26^∗^
Pain	0.13^#^	0.10^#^	−0.09^#^	−0.25^#^	0.26^#^	0.10^#^	−0.31^#^	0.02^#^	—	0.27^∗^
Function	0.03^#^	0.13^#^	−0.21^#^	−0.52^#^	−0.03^#^	−0.09^#^	−0.27^#^	0.13^#^	0.52^#^	—

*Note*. ^∗^Data in the right triangular region reflects pre-APA treatment; ^#^data in the left triangular region reflects the mean score change from pre- to post-APA treatment. CGRP: calcitonin gene-related peptide.
